# The Role of the Nuclear Receptor FXR in Arsenic-Induced Glucose Intolerance in Mice

**DOI:** 10.3390/toxics11100833

**Published:** 2023-10-01

**Authors:** Yifei Yang, Yun-Chung Hsiao, Chih-Wei Liu, Kun Lu

**Affiliations:** Department of Environmental Sciences and Engineering, University of North Carolina, Chapel Hill, NC 27599, USA

**Keywords:** arsenic, FXR, glucose tolerance, diabetes, glucose intolerance, proteomic, metabolomic, metabolic syndrome

## Abstract

Inorganic arsenic in drinking water is prioritized as a top environmental contaminant by the World Health Organization, with over 230 million people potentially being exposed. Arsenic toxicity has been well documented and is associated with a plethora of human diseases, including diabetes, as established in numerous animal and epidemiological studies. Our previous study revealed that arsenic exposure leads to the inhibition of nuclear receptors, including LXR/RXR. To this end, FXR is a nuclear receptor central to glucose and lipid metabolism. However, limited studies are available for understanding arsenic exposure-FXR interactions. Herein, we report that FXR knockout mice developed more profound glucose intolerance than wild-type mice upon arsenic exposure, supporting the regulatory role of FXR in arsenic-induced glucose intolerance. We further exposed mice to arsenic and tested if GW4064, a FXR agonist, could improve glucose intolerance and dysregulation of hepatic proteins and serum metabolites. Our data showed arsenic-induced glucose intolerance was remarkably diminished by GW4064, accompanied by a significant ratio of alleviation of dysregulation in hepatic proteins (83%) and annotated serum metabolites (58%). In particular, hepatic proteins “rescued” from arsenic toxicity by GW4064 featured members of glucose and lipid utilization. For instance, the expression of PCK1, a candidate gene for diabetes and obesity that facilitates gluconeogenesis, was repressed under arsenic exposure in the liver, but revived with the GW4064 supplement. Together, our comprehensive dataset indicates FXR plays a key role and may serve as a potential therapeutic for arsenic-induced metabolic disorders.

## 1. Introduction

Exposure to inorganic arsenic (i.e., arsenite, AsIII) in drinking water is a global public health threat. It was estimated that people living in nearly 108 countries and more than 230 million people are exposed to inorganic arsenic-contaminated water [1,2]. Inorganic arsenic was listed among the top ten environmental contaminants of public health concern by the World Health Organization (WHO) due to the increasing body of evidence linking chronic arsenic exposure to metabolic diseases such as type 2 diabetes mellitus. Several epidemiological studies have revealed a positive association between arsenic exposure and metabolic syndromes and even diabetes incidence [3]. The meta-analysis conducted in 2012, sponsored by the National Toxicology Program, concluded that there is limited to sufficient support for an association between arsenic and diabetes in populations with ≥150 μg-arsenic/L in drinking water [4]. To further reveal the mode of action, many in vitro and in vivo studies reported glucose intolerance [4,5,6,7], decreased insulin secretion [4,5,7,8], reduced visceral adiposity [5,9,10], and other metabolic disruptions. However, how arsenic uptake triggers molecular events that result in phenotypic metabolic syndromes remains ambiguous. Previous studies suggested that nuclear receptors (NRs), which are a superfamily of ligand-regulated transcription factors that account for many facets of physiology, were impaired due to arsenic toxicity. For instance, it was suggested that arsenic inhibited the transcriptional activation of the liver X receptor (LXR)/retinoid X receptor (RXR) heterodimers, thus impairing the excretion of cholesterol that led to atherosclerosis and diabetogenic phenotypes in animals [11,12,13].

The farnesoid X receptor belongs to the same nuclear receptor subfamily as LXR (i.e., the NR1H subfamily), and FXR also plays coordinating roles in the pathogenesis of diabetes. FXR, highly expressed in the liver and intestines, is a transcriptional sensor for endogenous bile acids [14]. Bile acids can be agonists or antagonists of FXR in a structure- and concentration-dependent manner [15,16,17,18]. For instance, the main species comprising 40% of the total bile acid pool in humans, chenodeoxycholic acid (DCA), is a FXR agonist, while the main family of bile acids in mice, muricholic acids (MCAs), are generally FXR antagonists [18,19]. The activation of FXR limits intracellular bile acid levels and overall results in bile acid elimination [20]. FXR also shows importance in regulating lipid and glucose homeostasis. Activation of FXR was observed to improve glucose tolerance by reducing hepatic gluconeogenesis and glycolysis. Previous in vitro investigations exposed several human cell lines (BEAS-2B, RWPE-1, A549, HUC, and HDF) or rat lung epithelial cells to arsenic and concluded that arsenic could induce aerobic glycolysis [21,22]. The authors observed molecular events including increased extracellular/intracellular lactate and elevated HIF-1A, which is an established glycolysis master regulator. However, how arsenic toxicity perturbs glycolysis in vivo and how it further develops into systemic glucose intolerance remains unknown. On the other hand, FXR activation leads to lower circulative lipid levels by suppressing de novo fatty acid synthesis, increasing fatty acid β-oxidation, and reducing cholesterol breakdown [23,24]. To sum up, FXR is a central homeostat for the energy utilization in our body, and its impairment can lead to undesired metabolic syndromes, especially those that are diabetogenic.

## 2. Materials and Methods

### 2.1. Animal Experiments and Glucose Tolerance Test

**Figure 1:**
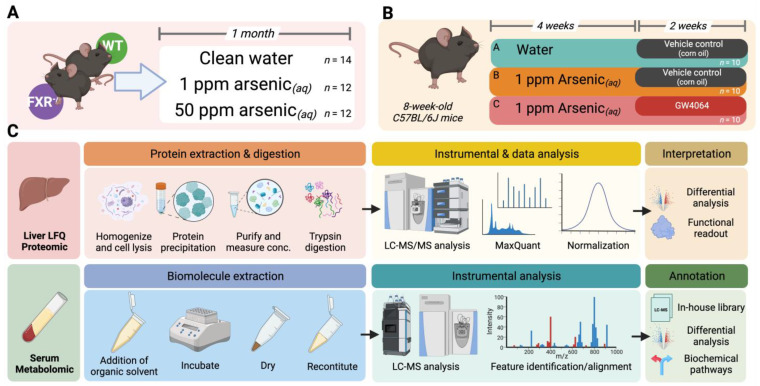
Schematic study design and experimental workflow for this study. (**A**) Dosing scheme of the first-stage animal experiment. Wild-type (WT) and FXR knockout (FXRKO) mice were exposed to drinking water with 0, 1, or 50 ppm arsenite for 1 month. (**B**) Dosing scheme of the second-stage animal experiment. WT mice were either exposed to clean drinking water for 6 weeks with the last 2 weeks orally administered with corn oil (Group A), exposed to drinking water containing 1 ppm arsenite for 6 weeks with the last 2 weeks orally administered with corn oil (Group B), or exposed to drinking water containing 1 ppm arsenite for 6 weeks with the last 2 weeks orally administered with GW4064 (Group C). (**C**) Experimental workflow of label-free quantitation of the liver proteome (upper panel) and nontargeted serum metabolomic profiling (lower panel).

All mice used in our studies were in the C57BL/6J background, male, purchased from the Jackson Laboratory (Bar Harbor, ME, USA), and housed in the animal facility of the University of North Carolina at Chapel Hill for a week before the experiments. All mice were provided a standard pelleted rodent diet and tap water ad libitum under the following environmental conditions: 22 °C, 40–70% humidity, and a 12:12 h light:dark cycle. There were two stages of animal experiments (Figure 1A,B). The drinking water provided to mice in the animal facility was processed with reverse osmosis water treatment and hyper-chlorination and stored in polycarbonate bottles with stainless steel sipper tubes. The drinking water itself contained arsenic levels lower than the detection limit of a standardized inductively coupled plasma mass spectrometry assay The water containing arsenite was prepared by dissolving sodium arsenite (NaAsO_2_, ≥98% purity) provided by Pfaltz & Bauer (Waterbury, CT, USA). For the first stage, wild-type (WT) and FXR knockout (FXRKO) mice were fed with clean drinking water (N = 14), drinking water with 1 ppm arsenite, or drinking water (N = 12) with 50 ppm arsenite (N = 12) for 1 month before the glucose tolerance test (GTT). For the second stage, the mice were randomly assigned into three groups (Groups A, B, and C, N = 10 per group). Group A mice were supplied with 6 weeks of clean drinking water, with the last 2 weeks orally administered with corn oil. Mice in Groups B and C were supplied with 6 weeks of drinking water containing 1 ppm arsenite, with respective oral administrations of corn oil and 30 mg/Kg-BW GW4064 dissolved in corn oil for the last two weeks. The oral administration of GW4064 (and corn oil solely) was practiced two times per day, as suggested by previous studies [25,26,27].

The mice were fasted for 14 h (18:30 from the previous day to 08:30) before the GTT. The tail snip blood of each mouse was adhered to a glucose test strip installed on a glucose meter (Contour Parsippany, Parsippany-Troy Hills, NJ, USA) at 0, 15, 30, 45, 60, 90, and 120 min after a 2 g/Kg-BW glucose administration. The mice were necropsied to collect their tissues and serum after euthanasia by carbon dioxide at the end of exposure. The animal use in this study was approved by the University of North Carolina at Chapel Hill Institutional Animal Care and Use Committee in accordance with the National Institutes of Health guidelines for the care and use of laboratory animals (with the approval number 22-191.0-B).

### 2.2. Label-Free Quantitation of the Hepatic Proteome

The overall analytical and experimental design is illustrated in Figure 1C. The methods for sample preparation and instrumental analysis of the hepatic proteome followed previous protocols and were thoroughly described in the Supporting Information [28]. In brief, 30 mg of liver tissue from each mouse was lysed, homogenized, and precipitated for protein. The protein lysates were purified and measured for concentration by a bicinchoninic acid (BCA) protein assay. Proteins from each sample were aliquoted to contain equivalent amounts, sequentially reduced, alkylated, and trypsin-digested. The peptide digest was then desalted by solid-phase extraction (SPE) and concentrated by SpeedVac^®^. The final peptide solution reconstituted to 0.5 mg/mL with 0.1% formic acid (aq) was injected (6 μL) into an Ultimate3000 RSLCnano system coupled to a Q-Exactive HF Orbitrap mass spectrometer through an EASY-Spray ion source for the nanoLC-nanoESI-MS/MS analysis. The nanoLC-MS/MS data for hepatic peptides were analyzed using MaxQuant ver. 2.3.1.0 against the Swiss-Prot mouse protein database to map and quantify the proteome.

### 2.3. Serum Nontargeted Metabolomics

The protocol for the MS-based metabolomic method applied in this study has been described previously, and the detailed procedures are provided in the Supporting Information [4,5,6,7,8,28,29,30,31,32]. In brief, 20 μL of serum from each mouse was extracted for metabolites, and the reconstituted metabolites were injected into a Thermo Fisher Scientific (Waltham, MA USA) Vanquish UHPLC coupled to a Q-Exactive Orbitrap mass spectrometer connected to a heated electrospray ionization (HESI) source for LC-MS analysis. Briefly, the extracted serum metabolites were recorded for their retention time (RT) and *m*/*z* in high-resolution. The molecular signatures (i.e., RT-*m*/*z* combinations) were deconvoluted, aligned across samples, and matched against the in-house LC-MS to annotate the biochemicals detected.

### 2.4. Differential Analysis

Differential analysis was conducted for the GTT, hepatic proteome, and serum metabolome. For the GTT, a group-wise F test was conducted to examine if differences existed among groups. Protein- or feature-wise student *t* test or F test after log-transforming the data was applied for the differential analysis of the proteomic or metabolomic datasets, and the *p*-values were adjusted by the Benjamin–Hochberg method (as *q*-values). A significant difference was defined as a 1.5-fold change in either direction with *q* < 0.05. In cases where a group-wise F test indicates a significant difference, a post-hoc pairwise Student *t* test that adjusts the *p*-value with the Benjamin–Hochberg procedure is subsequently used to suggest the origin of the difference among groups.

## 3. Results

### 3.1. FXR Is a Key Player in Arsenic-Induced Glucose Intolerance

To examine if FXR was involved in arsenic-induced glucose intolerance, we supplied drinking water with 0, 1, or 50 ppm arsenic to FXRKO and WT mice (Figure 1A). The body weights were lower in FXRKO than in WT mice (Figure S1), which was consistent with previous reports [33]

**Figure 2:**
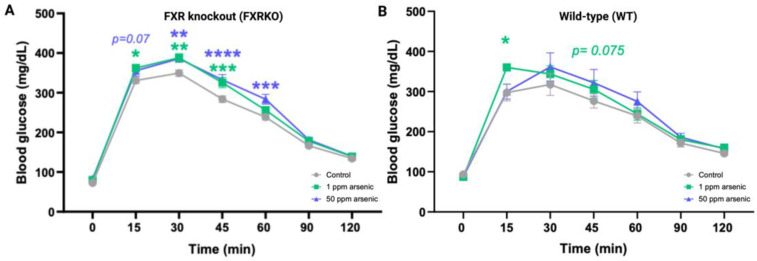
More profound glucose intolerance by arsenite was observed in FXR knockout (FXRKO) mice than in wild-type (WT) mice for the 1-month exposure to arsenite. (**A**) GTT resulted from FXRKO mice exposed to drinking water containing 0 (grey, N = 14), 1 (green, N = 12), or 50 (purple, N = 12) ppm arsenite for 1 month. (**B**) GTT resulted from WT mice exposed to drinking water containing 0 (grey, N = 5), 1 (green, N = 5), or 50 (purple, N = 5) ppm arsenite for 1 month. Error bars are illustrated for the standard error of the mean. A post-hoc pairwise *t* test was conducted, and the group-wise F test resulted in a significant difference. * *q* < 0.05, ** *q* < 0.01, *** *q* < 0.001, **** *q* < 0.0001 in a post-hoc pairwise *t* test against the control mice without arsenite exposure.

Arsenic-induced glucose intolerance was observed in both WT and FXRKO mice (Figure 2) in the GTT, but the intolerance was more profound for the FXRKO mice (Figure 2A). WT mice exposed to 1 ppm arsenic compared to the unexposed WT mice had significantly higher glucose levels in the GTT at the 15-min timepoint (Figure 2B). In contrast, FXRKO mice with exposure to 1 ppm arsenic compared to non-exposure resulted in higher glucose levels from 15 to 45 min after initiating the GTT; furthermore, exposure to 50 ppm arsenic resulted in higher glucose levels than non-exposure in the GTT test from 30 to 60 min (Figure 2A).

**Figure 3:**
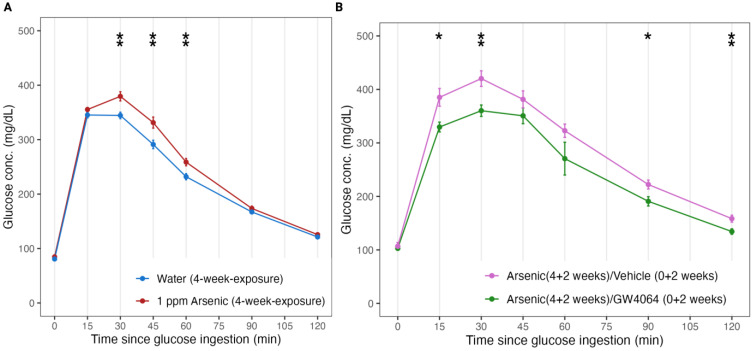
FXR agonist GW4064 alleviated arsenite-induced glucose intolerance in mice. (**A**) Glucose tolerance test (GTT) results in mice exposed to 4 weeks of drinking water with (Group B and Group C, N = 20 combined, colored red) and without (Group A, N = 20, colored blue) 1 ppm arsenite. (**B**) GTT results in mice with (Group C, N = 10, colored green) and without (Group B, N = 10, colored pink) the additional oral administration of GW4064 for the last 2 weeks of the 6-week exposure of 1 ppm arsenite via drinking water. Error bars are illustrated for the standard error of the mean. * *p* < 0.05, ** *p* < 0.01.

To investigate how FXR was involved in arsenic-induced glucose intolerance, we designed the second stage of the animal experiment to inspect if the introduction of a FXR agonist could reverse the arsenic-induced glucose intolerance (Figure 1B). Before the administration of GW4064, the chosen FXR agonist, we observed explicit glucose intolerance following 4 weeks of arsenic exposure (Figure 3A). Since then, with the supplement of GW4064 dissolved in corn oil, the mice improved in glucose tolerance compared to the mice fed only with the vehicle control (Figure 3B). For instance, Group C mice had lower blood glucose levels at the 15 and 30 timepoints (329.9 ± 28 and 360.2 ± 33) in the GTT compared to Group B mice (385.2 ± 52 and 420 ± 46) (*p* = 0.01 and 0.004). The glucose tolerance performance of arsenic-exposed mice leaned towards glucose homeostasis in mice unexposed to arsenic (Figure S2).

### 3.2. Impact of Arsenic Exposure and the Reverse Effect of FXR agonists in the Hepatic Proteome

**Figure 4:**
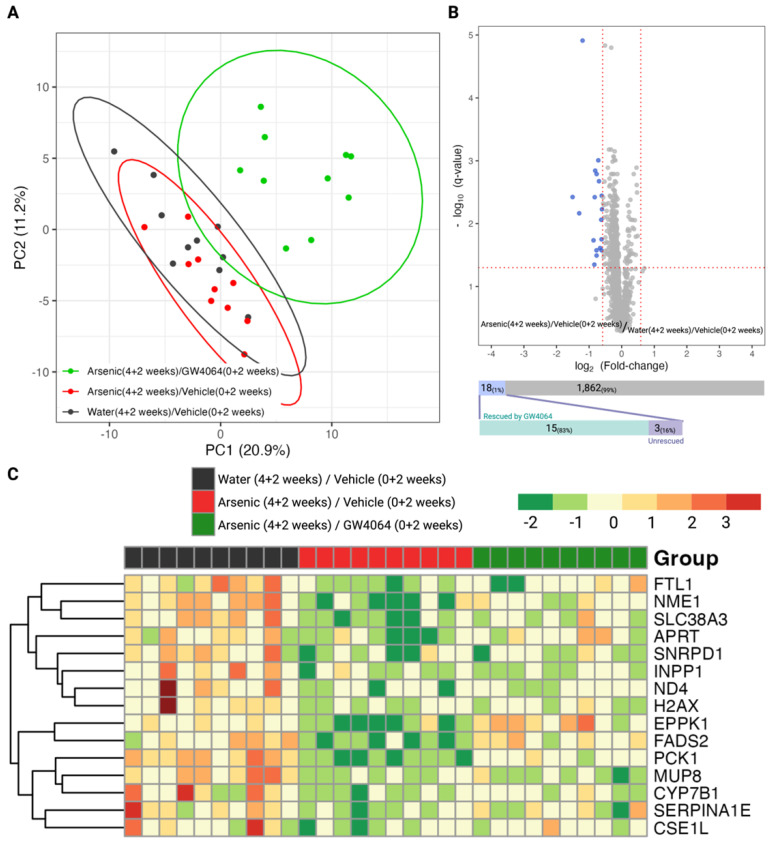
Exploratory and differential analysis of the label-free quantitation of the hepatic proteome show arsenite dysregulations and the alleviating effect of GW4064. (**A**) Clustering result from principal component analysis of the 1880 hepatic proteins profiled in mice from the second stage animal experiment. (**B**) Differential analysis to recognize dysregulated hepatic proteins by arsenite exposure. The x- and y-axes record the binary logarithm value of fold change and the *q*-value of protein-wise Student *t*-test between the hepatic proteins of mice exposed to drinking water with and without 1 ppm arsenite (Group B versus Group A). The *q*-value for each protein resulted from adjusting for the false discovery rate with the Benjamin–Hochberg procedure and the p-value. The significant difference was defined as a *q*-value < 0.05 and a fold change > 1.5 in either direction. Hepatic proteins with significantly higher or lower expression in the arsenite-treated group were respectively colored red and blue. The number of dysregulated proteins was annotated in the upper bar under the volcano plot, with the lower bar indicating the number of dysregulated proteins rescued by the supplement GW4064. (**C**) Heatmap of the 15 arsenite-dysregulated hepatic proteins rescued by GW4064 in mice without arsenite and GW4064 (Group A, black), with arsenite but without GW4064 (Group B, red), and with both arsenite and GW4064 (Group C, green) exposure. Hepatic protein levels were standardized and converted into color, with a gradient from red to green indicating high to low levels.

We observed that FXR was involved in the arsenic-induced glucose intolerance; thus, we further profiled the hepatic proteome and serum metabolome for the mice in the Stage 2 animal experiment to elucidate the molecular events (Figure 1C). We were able to detect 1180 hepatic proteins with satisfying instrumental quality (Figure S3), and the corresponding principal component analysis is presented in Figure 4A. The clustering result indicates that the FXR agonist GW4064 globally alternated the expression of hepatic proteins, reflecting a distinctive cluster compared to the groups without GW4064 administration. We further examined what hepatic proteins were dysregulated by arsenic exposure (by comparing Groups B against A), and 18 proteins were down-regulated by arsenic exposure (Figure 4B). We were interested in knowing if the down-regulation of these proteins was associated with FXR; thus, we investigated if the down-regulations were alleviated in Group C, in which the mice were supplemented with GW4064. Most of the proteins (15/18, 83%) had alleviated arsenic-induced down-regulation with the administration of GW4064, which is shown in the heatmap provided in Figure 4C.

### 3.3. Shift of Serum Metabolites by Arsenic Exposure and How FXR agonists Can Rescue Alterations

**Figure 5:**
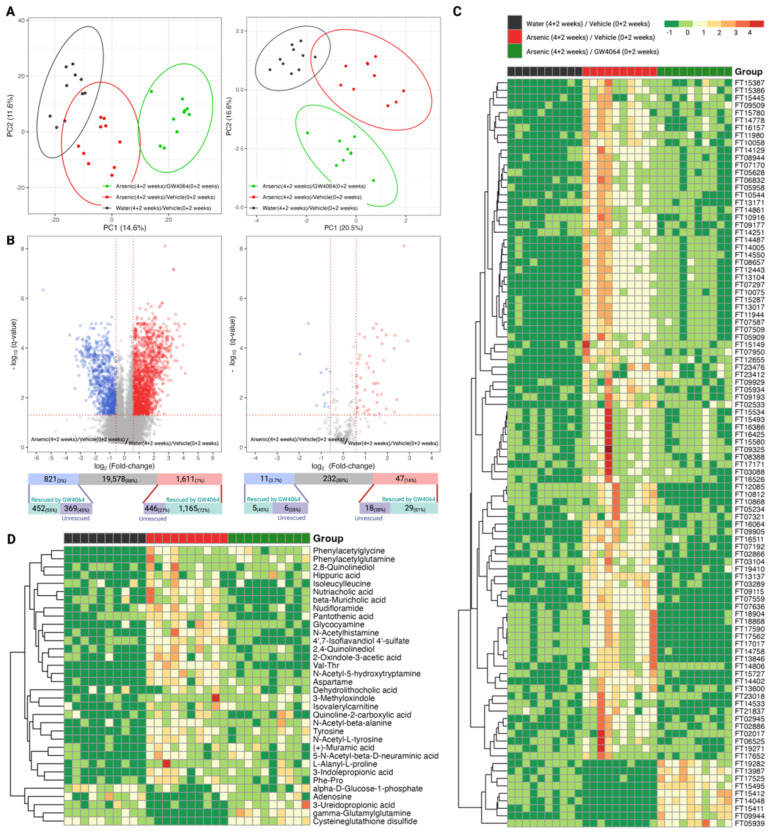
Exploratory and differential analysis of the nontargeted profiling of the serum metabolome show arsenite dysregulation and the alleviating effect of GW4064. (**A**) Clustering result from principal component analysis of the 22,010 molecular features (left panel) and 290 annotated compounds (right panel) from the serum of mice from the second stage of the animal experiment. (**B**) Differential analysis to recognize dysregulated serum metabolites by arsenite exposure using the complete molecular features (left panel) or the total annotated compounds (right panel). The x- and y-axes record the binary logarithm value of fold change and the *q*-value of feature-wise Student *t*-test between the serum metabolites of mice exposed to drinking water with and without 1 ppm arsenite (Group B versus Group A). The *q*-value for each feature was determined by adjusting for the false discovery rate with the Benjamin–Hochberg procedure and the *p*-value. The significant difference was defined as a *q*-value < 0.05 and a fold change > 1.5 in either direction. Serum metabolites with significantly higher or lower levels in the arsenite-treated group were respectively colored red and blue. The number of dysregulated metabolites was annotated in the upper bar under the volcano plot, with the lower bar indicating the number of dysregulated proteins rescued by the additional administration of GW4064. (**C**,**D**) Heatmap of the top 100 molecular features that had that largest alleviating effect of GW4064 in arsenite dysregulation (**C**) and the annotated compounds that had alleviating effect of GW4064 in arsenite dysregulation (**D**) in mice without arsenite and GW4064 (Group A, black), with arsenite but without GW4064 (Group B, red), and with both arsenite and GW4064 (Group C, green) exposure. Serum metabolite levels were standardized and converted into color, with a gradient from red to green indicating high to low levels.

Since FXR is a key homeostat for energy homeostasis, especially in the utilization of carbohydrates and lipids, we monitored the metabolic landscape in the serum of mice exposed to arsenic and mediated by GW4064 (Figure 1C). With the strength of the high-resolution nontargeted metabolomic workflow, we were able to detect 22,010 molecular signatures among the serum of the three groups of mice with assured instrumental performance and stability (Figures S4 and S5). These 22,010 features can be queried against our in-house LC-MS library, resulting in 290 characterized metabolites. The overall metabolic alterations attributed to arsenic and GW4064 can apparently be observed, either by using the total molecular features or those successfully annotated with a chemical identity (Figure 5A). We focused on the metabolites dysregulated by arsenic exposure. Among the 22,100 features, 2432 (11%) of them were significantly altered by arsenic exposure (1.5-fold change in either direction and *q* < 0.05), with 1611 (7.3%) and 821 (3.7%) features, respectively, being up- and down-regulated. For the annotated metabolites, there were dysregulations in 58 (20%) compounds, of which 47 (16%) and 11 (3.7%) compounds showed significantly increased and decreased levels, respectively.

To answer whether FXR belongs to the network of these arsenic-induced alterations, we investigated if the dysregulations were alleviated for the 2432 features and the 58 compounds. For the 2432 arsenic-dysregulated features, 72% (1165/1611) of the up-regulated and 55% (452/821) of the down-regulated features show milder dysregulation under arsenic exposure with the supplement of GW4064. The alleviating effects were consistent for the 58 dysregulated compounds, of which 61% (29/47) of the elevated and 45% (5/11) of the reduced metabolites showed a relieved imbalance when GW4064 was additionally supplied under arsenic intake. The heatmaps in Figure 5C illustrated how GW4064 alleviated dysregulation caused by arsenic for the top 100 features with the highest rescuing effect. The extent of the rescuing effect was calculated by the absolute difference between the fold-change among the 2432 arsenic-dysregulated features of the following two comparisons: Group B versus Group A and Group C versus Group A. Using the same approach, the alleviation of GW4064 in arsenic exposure can be visualized for the 34 “rescued” compounds (29 up- and 5 down-regulated, combined) in Figure 5D.

### 3.4. Arsenic-Induced Dysregulation of Protein in Glucose and Lipid Metabolism Is Associated with FXR

**Figure 6:**
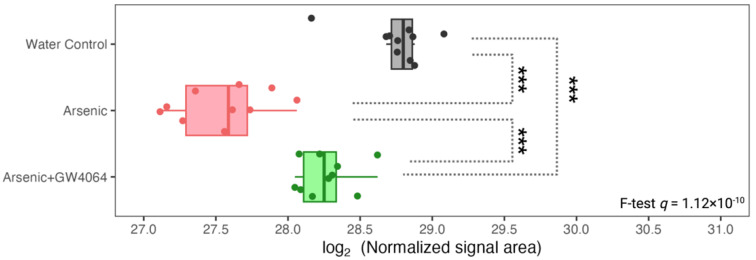
Expression of hepatic PCK1, a key player in gluconeogenesis, was lower under arsenite exposure, but the reduction was alleviated by GW4064. The x-axis indicates the binary logarithm value of the hepatic PCK1 level. The y-axis is labeled with the different groups. The *q*-value of PCK1 in the protein-wise F test is labeled. The pairwise *t*-test result is asterisked to show pairwise differences, with the *p*-value adjusted by the Benjamin–Hochberg procedure. *** *q* < 0.001.

**Figure 7:**
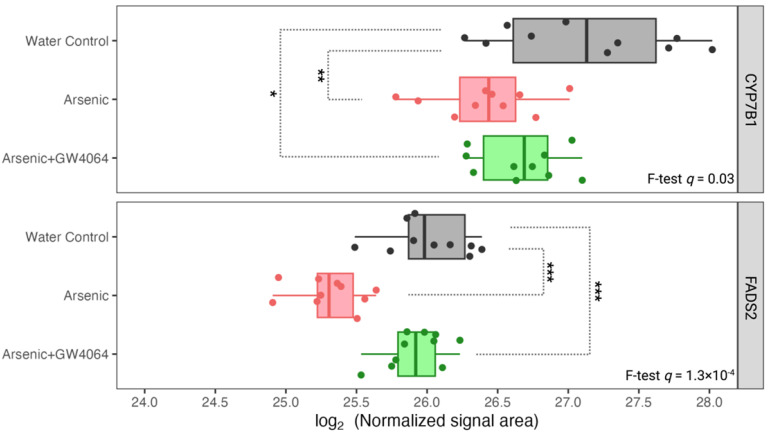
Expression of hepatic proteins related to cholesterol and lipid metabolism, CYP7B1 and FADS2, was reduced under arsenite exposure, but the decreases were smaller with the GW4064 supplement. The x-axis indicates the binary logarithm value of the hepatic protein level. The y-axis is labeled with the different groups. The *q*-values of CYP7B1 and FADS2 in the protein-wise F test are labeled. The pairwise *t*-test results are asterisked to show pairwise differences, with the *p*-value adjusted by the Benjamin–Hochberg procedure. * *q* < 0.05, ** *q* < 0.01, *** *q* < 0.001.

**Figure 8:**
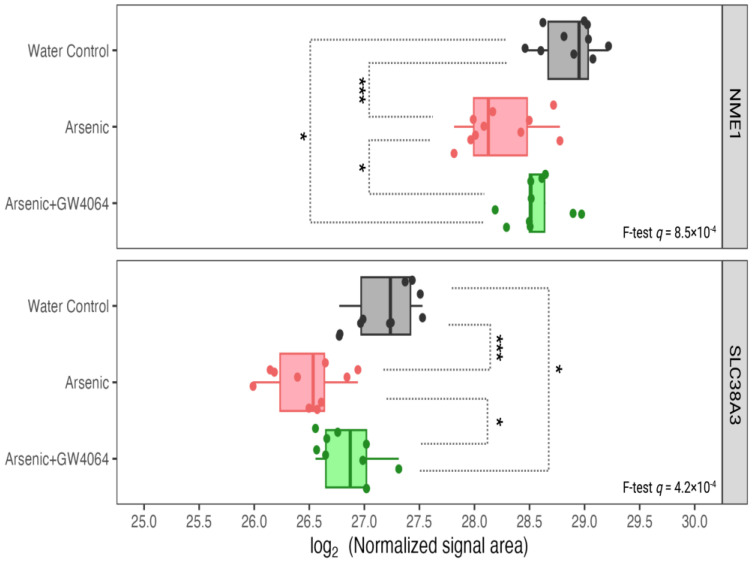
Expression of other hepatic proteins involved in glucose metabolism, such as NME1 and SLC38A3, was reduced under arsenite exposure, but the decreases were smaller with the GW4064 supplement. The x-axis indicates the binary logarithm value of the hepatic protein level. The y-axis is labeled with the different groups. The *q*-values of NME1 and SLC38A3 in the protein-wise F test are labeled. The pairwise *t*-test results are asterisked to show pairwise differences, with the *p*-value adjusted by the Benjamin–Hochberg procedure. * *q* < 0.05, *** *q* < 0.001.

Proteins highly involved in glucose and lipid metabolism can be found in the 15 proteins down-regulated by arsenic exposure and subsequently reversed by the supplement GW4064. For instance, hepatic PCK1, a protein responsible for both gluconeogenesis and glyceroneogenesis, had the highest level in Group A (water control) mice, with the expression in arsenic-exposed Group B mice severely reduced (fold-change 0.43, post-hoc *p* = 5.7 × 10^−11^) (Figure 6). With the remedy of GW4064, although the level of PCK1 did not reach the level shown in mice unexposed to arsenic, it significantly increased (Group C versus Group B increased 1.62, post-hoc *p* = 1.4 × 10^−5^), and the down-regulation induced by arsenic was alleviated. The same remedying effect of GW4064 on arsenic exposure can be observed for CYP7B1 and FADS2, two proteins that facilitate cholesterol biotransformation and lipid metabolism (Figure 7). We observed other proteins that were postulated as regulators of glucose metabolism with the dysregulation by arsenic and subsequent remedy by GW4064. For instance, NME1 and SLC38A3 both show critical down-regulation after arsenic exposure, but the introduction of GW4064 alleviated the reductions for both of these proteins (Figure 8).

## 4. Discussion

To understand if FXR is involved in the metabolic syndromes attributed to arsenic exposure, we exposed FXRKO and WT mice to arsenic in drinking water (Figure 1A) and observed more severe glucose intolerance in FXRKO mice (Figure 2), which served as the initial evidence of the involvement of FXR in arsenic metabolic disturbance. When the WT mice were exposed to arsenic and supplemented with GW4064 (synthetic FXR agonist), there was an improvement in glucose tolerance. Many of the dysregulations induced by arsenic exposure in hepatic proteins and serum metabolites were reversed by GW4064 (Figure 4 and Figure 5). The “rescued” hepatic proteins include key mediators of glucose and lipid metabolism (e.g., PCK1, CYP7B1). Reversed serum metabolites include bile acids, fatty acid species, and compounds involved in tryptophan/indole metabolism.

Previous research reported that FXR is essential for normal glucose homeostasis, as FXRKO mice showed elevated serum glucose and impaired insulin sensitivity [34,35,36]. Our comparison of the GTT between FXRKO and WT mice was consistent with previous observations (Figure 2). Arsenic exposure was reported as a risk factor for glucose intolerance as well, but the underlying mechanism remained ambiguous, and the available literature focused more on LXR and RXR [11,12,13]. Our data support the notion that FXR is involved in arsenic-induced glucose intolerance, as FXRKO mice show deteriorated glucose intolerance compared to WT mice (Figure 2). The activation of FXR is known to maintain glucose normality by suppressing postprandial gluconeogenesis. We hypothesize that arsenic may suppress FXR expression, and the suppression can contribute to phenotypic glucose intolerance. To more comprehensively prove our theory, we introduced a FXR agonist into the arsenic exposure scheme to see if a reactivation of the suppressed FXR (by arsenic) could reverse the glucose intolerance, and the results were positive (Figure 3). This experiment evidenced that FXR is a key mediator in arsenic-induced glucose intolerance. We sought to reveal the underlying molecular events by monitoring the liver proteomics and serum metabolomics of mice fed arsenic (Group B), arsenic and GW4064 supplement (Group C), and their control (clean water and no supplement, Group A).

We were able to recognize hepatic proteins that were down-regulated by arsenic exposure, and most of them (83%) showed alleviated dysregulation in the mice supplemented with GW4064 (Figure 4). These proteins can be considered highly associated with FXR activity; when arsenic impaired FXR, these proteins were consistently down-regulated as well. As a result, by understanding the functions of these proteins and their interaction with FXR, we revealed possible mechanisms for how arsenic impairs FXR and thus induces metabolic disruption. Arsenic significantly reduced the level of hepatic PCK1, and the reduction was alleviated after the introduction of GW4064 (Figure 6). The phosphoenolpyruvate carboxykinase (PCK) protein family has been exclusively recognized as a critical enzyme in gluconeogenesis, especially in the liver and kidney [37]. PCK1, the cytosolic form of PCK that constitutes almost 95% of the total PCK in the murine liver, catalyzes the reaction of oxaloacetate (OAA) into phosphoenolpyruvate (PEP), which is the initial reaction of the four rate-limiting steps of gluconeogenesis [37,38]. PCK1, with its central role in regulating gluconeogenesis and its other secondary function, is essential to survival, as mice without PCK1 suffer from severe hypoglycemia after birth and die [38]; therefore, PCK1 is a candidate diabetes and obesity gene [39,40]. Among the three groups of mice (water control, arsenic, and arsenic with GW4064 supplement), arsenic exposure resulted in the lowest hepatic PCK1, followed by arsenic exposure supplemented with GW4064 (Figure 6). The results are inconsistent with previous studies. Previous research showed that FXR activation suppressed PCK1, as the administration of GW4064 eliminated PCK1 expression [41,42], which is consistent with our observation. On the other hand, 16 weeks of 3 mg/L arsenic exposure via drinking water were shown to increase hepatic PCK1 in C57BLKS/J mice [43]. The dosing duration and concentration were higher for this previous study, and C57BLKS/J, a diabetes-prone strain, compared to C57BL/6J mice, developed more severe diet-induced atherosclerotic lesions [44]. Our data show that arsenic significantly decreases hepatic PCK1 levels. Difficulties remained in understanding why our results differed from the previous report, as the dosing scheme and mouse strain were different. The glucose intolerance that resulted from arsenic exposure led to glucose accumulation in the circulation and limited gluconeogenesis, which can explain why we observed a down-regulation of PCK1 under arsenic exposure.

Our observation that arsenic exposure reduced hepatic CYP7B1 is consistent with previous studies with various arsenic doses in mice [45,46,47]. CYP7B1 is one of the cytochrome P450 (CYP) monooxygenases that is involved in the biosynthesis of bile acids, oxysterols, and steroid hormones by consuming cholesterol. After cholesterol is converted into 27-hydroxycholesterol by CYP27A1, CYP7B1 further facilitates its biotransformation to 3β,7α-dihydroxy-5-cholestenoic acid, which eventually forms chenodeoxycholic acid (CDCA) [48]. We may suspect that, with the reduction of CYP7B1 under arsenic exposure, there were decreased bile acid levels as well. However, in our serum metabolomic analysis, we observed elevated serum levels of β-muricholic acid, nutriacholic acid, and dehydrolithocholic acid (Figure 5D). The biosynthesis of different primary and secondary bile acids was facilitated by very distinctive proteins [49,50], and previous studies have shown that arsenic regulates bile acids in a structural and gut microbiota-related manner [51]. Our result of higher serum β-muricholic acid levels after arsenic exposure (Figure 5D) was consistent with previous results [51].

FADS2 is another protein accountable for lipid metabolism that was observed to be reduced in the liver after arsenic exposure (Figure 7). After the administration of GW4064, the arsenic-induced suppression of FADS2 expression was alleviated. FADS2, a member of the fatty acid desaturase (FADS) family, regulates physiological lipids by converting saturated fatty acids into unsaturated and polyunsaturated fatty acids (UFAs and PUFAs) [52]. The specific reactions FADS2 catalyzes can be classified into the PUFA type and the sebaceous type. The PUFA-type reaction converts essential fatty acids of the n-6 family (linoleate) and n-3 family (α-linoleate) into γ-linoleate and stearidonate, respectively [53]. The sebaceous-type reaction introduces an additional cis double bond to the saturated palmitate and results in mono-unsaturated sapienate. PUFA deficiency is known to induce hepatic steatosis (fatty liver), and previous studies showed that the ablation of FADS2 in mice exacerbates hepatic triacylglycerol and cholesterol accumulation [54]. The association between the reductions of FXR and FADS2 after arsenic exposure was not surprising, as FXR suppression is known to induce fatty acid uptake, lipogenesis, and repress triglyceride clearance. However, there is little previous research on the co-expression and interaction between FXR and FADS. We identified other arsenic-induced down-regulations of hepatic proteins that were associated with glucose homeostasis in previous studies. For instance, high glucose was considered a repressing factor for NME1 [55], and we observed lowered NME1 hepatic expression in glucose-intolerant arsenic-exposed mice (Figure 8). It is also believed that dietary-derived amino acids can modulate the utilization and disposal of blood glucose by acting as mediators of insulin signaling [56,57], and the SLC38 family is the most responsible solute carrier (SLC) for glutamine transportation. The reduction of SLC38A3 was observed after arsenic exposure (Figure 8). The down-regulations of NME1 and SLC38A3 after arsenic exposure can both be alleviated after the supplementation of GW4064.

There are limitations to this study. Both the hepatic proteome and serum metabolome were cross-sectional profiles of the mice. There are physiological dynamics of these proteins and metabolites that can differ in fasting, postprandial, or intra-prandial periods. Our study provides a set of preliminary data to highlight the existing interaction between arsenic exposure and FXR function and their subsequent molecular dysregulations. Although our described protein interplay implied what mechanisms underlie the glucose intolerance caused by arsenic exposure, more studies including the biomonitoring of different tissues and different molecular targets are needed to approximate the molecular cascades of arsenic in triggering diabetogenic outcomes. In addition, though our serum metabolomic workflow provides unbiased and global screening of the metabolites imbalanced after arsenic exposure, a stricter criterion was applied to ensure the reliability of the annotations. More biomolecules may be profiled in the future for a better understanding of the arsenic-FXR complex. Regardless, in this study, we demonstrate that FXR plays a key role in arsenic-induced glucose intolerance. Activation with a FXR agonist significantly reduces arsenic-induced glucose intolerance and associated dysregulation of hepatic proteins and serum metabolites, highlighting that FXR serves as a potential target for the treatment of arsenic-induced diabetes.

## 5. Conclusions

Arsenic exposure has shown in vivo diabetogenic effects, such as glucose intolerance. Previous studies focused on the disruption caused by arsenic on other receptors (LXR, RXR), with less investigation dedicated to understanding how arsenic interacts with FXR. We argue that ingested arsenic could impair FXR functioning, resulting in imbalanced energy dynamics. In this study, we first observed more profound arsenic-induced glucose intolerance in FXRKO than in WT mice, which supported our hypothesis of the interaction between FXR and arsenic. We then exposed WT mice to arsenic for 6 weeks and supplemented GW4064, a synthetic FXR agonist, in the last two weeks of exposure and observed improved glucose tolerance. We found that the down-regulation of hepatic proteins by arsenic can be reversed with the GW4064 supplement. The “rescued” proteins contained regulators of glucose and lipid metabolism (PCK1, CYP7B1, FADS2, NME1, SLC38A3). The alleviating effect of GW4064 on arsenic-induced dysregulation of serum metabolites was also observed. These data serve as evidence that the diabetogenic effects, such as glucose intolerance, caused by arsenic exposure are facilitated through mechanisms including FXR dysregulation.

## Data Availability

Data are available from the corresponding author upon reasonable request.

## Supplementary Materials

The following supporting information can be downloaded at: https://www.mdpi.com/article/10.3390/toxics11100833/s1, Figure S1: Body weights of the different groups of mice in the first stage animal experiment; Figure S2: Blood glucose level throughout glucose tolerance test for the second stage of animal experiments; Figure S3: Signal distribution of the label-free quantitation of the hepatic proteome; Figure S4: Signal distribution of the nontargeted profiling of the serum metabolome; Figure S5: Signal areas of the stable isotope-labeled standards spiked in each sample for serum metabolomic [58,59,60,61,62,63,64,65,66,67,68,69,70,71].
