# Supplementary material for: The Role of the Nuclear Receptor FXR in Arsenic-Induced Glucose Intolerance in Mice
Source: Toxics. 2023 Oct 1;11(10):833. doi: 10.3390/toxics11100833 (PMC10611046; doi:10.3390/toxics11100833)
Supplement: Supplementary file 1 [file toxics-11-00833-s001.zip › toxics-2587057-supplementary.pdf]

Supporting Information for

The Role of FXR in Arsenic-induced Glucose Intolerance in Mice

Yifei Yang[1#], Yun-Chung Hsiao[1#], Chih-Wei Liu[1], Kun Lu[1*]

* Corresponding author

# Contributed equally

Affiliations:

[1] Department of Environmental Sciences and Engineering, University of North Carolina, Chapel Hill, NC 27599

Corresponding Author: Dr. Kun Lu

Address: Department Environmental Sciences and Engineering, University of North Carolina at Chapel Hill, North Carolina 27599, United States

Email: kunlu@unc.edu

Telephone: (+1) 919-966-7377

Keywords: arsenic, FXR, glucose tolerance, diabetes, glucose intolerance, proteomic, metabolomic, metabolic syndrome

Table of Content

Extended methodology for nanoLC-nanoESI-MS/MS proteomic analysis for liver tissue………….………… 3

Extended methodology for serum LC-MS(/MS) metabolomic analysis …………..……………………………… 5

Figure S1. Body weights of the different groups of mice in the first stage animal experiment……………….. 7

Figure S2. Blood glucose level throughout glucose tolerance test for the second stage of animal experiments. 8

Figure S3. Signal distribution of the label-free quantitation of the hepatic proteome. …………………………9

Figure S4. Signal distribution of the nontargeted profiling of the serum metabolome. ……………………… 10

Figure S5. Signal areas of the stable isotope-labeled standards spiked in each sample for serum metabolomic. 11

Reference ………………………………………………………………………………….…………..………… 12

Extended methodology for nanoLC-nanoESI-MS/MS proteomic analysis for liver tissue

Hepatic protein extraction. Liver tissue (~30 mg) from each mouse were lysed and homogenized in lysis buffer (4% SDS, 100 mM DTT, 100 mM Tris pH 7.5, phosphatase inhibitors cocktail 2 and 3, and protease inhibitor cOmplete® at manufacturer suggested ratios) by the mechanical disruption of TissueLyzer for 10 minutes (50 Hz). The lysed tissue samples were centrifuged at 16,000 ×g for 10 minutes at 4°C, and the supernatant was collected for protein precipitation. Protein lysates were precipitated using acidified acetone/ethanol (5-fold volume, 50:50 (v/v), with 0.1% acetic acid) at -20 °C for overnight. Proteins were spun down by centrifugation at 16,000 ×g for 10 min at 4 °C, and washed with ice-cold acetone for three times. The protein pellets were then re-suspended in 6 M urea in 50 mM ammonium bicarbonate (pH 8.0) and measured for concentration by BCA.

Protein digestion. The proteins were sequentially reduced and alkylated by dithiothreitol (10 mM) and iodoacetamide (40 mM) for 1 hr at 37 °C, and then the alkylation was quenched by adding dithiothreitol for 15 min at 37°C. The protein solution was further diluted with 50 mM ABC to reduce urea concentration (<1 M) before digestion by trypsin (1:100 enzyme:protein ratio) in the present of 1 mM $CaCl_2$ at 37 °C with gentle shaking for overnight. Finally, the digestion was terminated by acidifying with formic acid (FA) before solid-phase extraction (SPE) desalting clean-up.[1,2] The desalted peptides were dried in vacuum and reconstituted with 0.1% FA for peptide concentration measurement (NanoDrop UV-Vis spectrophotometer, A_{205} (scope mode), Thermo Fisher Scientific) before mass spectrometry analysis.

Instrumental analysis. The LC-MS/MS analysis was performed using an UltiMate 3000 RSLCnano system coupled to a Q Exactive HF Orbitrap mass spectrometer through an EASY-Spray ion source (Thermo Fisher Scientific). A binary solvent system (solvent A: 0.1% FA in water; solvent B: 0.1% FA in acetonitrile) was used for peptide separation at a flow rate of 250 nL/min. Peptides (0.5 µg/µL, 6 µL) were separated on a PepMap C_{18} analytical column (2 µm particle, 50 cm × 75 µm i.d., Thermo Fisher Scientific). LC separation was performed using the following gradient: held at 2% B for 4 min, from 2% to 40% B in 89 min, 40% to 95% B in 0.1% min, held at 95% B for 10 min, 95% to 2% B in 0.1 min, and held at 2% B for 17 min for re-equilibrating column. MS and MS/MS spectra were acquired in profile mode using a data-dependent top-15 method and resolution for full MS scan (350 to 1,650 *m/z*) was set to 60,000 at *m/z* 200 with maximum fill time of 50 ms. Precursors were isolated with a window of 1.4 m/z and fragmented with higher-energy collisional dissociation (HCD, normalized collision energy of 27). Resolution for MS/MS spectrum was set to 30,000 at *m/z* 200 with maximum full time of 50 ms. The AGC (automatic gain control) targets for MS and MS/MS scans were 1×10^6 and 2×10^5, respectively. Precursor ions with unassigned, single, seven and higher charge states were excluded from fragmentation, and dynamic exclusion time was set to 20 s with peptide match function enabled.

Data processing for proteome. The LC-MS/MS raw files were analyzed using MaxQuant software version 2.3.1.0, and searched against Swiss-Prot mouse protein database (86,376 protein entries, downloaded on 06/26/2017) using the built-in Andromeda search engine.[3] Specific Trypsin/P was selected as the enzyme.

Cysteine carbamidomethylation was set as fixed modifications. Methionine oxidation and acetyl protein N-termini were set as variable modifications. False discovery rate (FDR) was set at 1% for protein and peptide level identification using a decoy database. Both unique and razor peptides were used for label free protein quantification. Other parameters were used as default settings for Orbitrap-type data. The search results (LFQ intensity) in proteinGroups.txt generated by MaxQuant were processed in Perseus software version 2.0.9.0.[4] Identified protein was represented as protein group in the database search output when the search algorithm identified a cluster of proteins with high sequence similarity or protein isoforms, which cannot be further differentiated on the basis of shared peptides. The potential contaminants, reverse hits and proteins only identified by modification site were excluded from the final identification list before further statistical analyses.

Extended methodology for serum LC-MS(/MS) metabolomic analysis

Sample preparation. Serum samples from each mouse were thawed and 20 μL was consumed to extract metabolites for each mice. Metabolite extraction was facilitated by adding 180 μL methanol containing stable isotope-labeled chemicals ([D_5]-glutamine, [D_2]-γ-aminobutyric acid, [D_3]-tryptophan, [D_2]-indole-3-propionic acid, [D_2]-indole-3-acetic acid, [D_{13}]-acetylcholine in 500 nM; [D_4]-serotonin in 250 nM; and [D_4]-kynurenic acid in 50 nM), and incubated under -20°C for 1 hr, followed by centrifugation under 15,000 ×g, 4°C for 10 minutes to collect the supernatant (150 μL). The supernatant was dried by SpeedVac® and reconstituted with 100 μL 2% acetonitrile in water.

Instrumental analysis. The LC-MS analysis was facilitated by a Thermo Fisher Scientific Vanquish UHPLC coupled to a Q Exactive mass spectrometer connected by a heated electrospray ionization (HESI) source, and the method applied was previously reported [5-7]. The serum analytes were injected (10 μL) into a Waters Acquity UPLC HSS T3 (reverse phase C18, 100 Å, 1.8 μm, 2.1 mm × 100 mm) analytical column controlled at 40 °C, with the mobile phase composed of water (A) and acetonitrile (B) both added with 0.1% formic acid at a flow rate of 0.4 mL/min. The 15-min-gradient for chromatographic separation was set as the following: 2% B from 0-1 min; 2%-15% B from 1-3 min; 15%-50% B from 3-6 min; 50%-98% B from 6-7.5 min; 98% B held from 7.5-11.5 min; 98%-2% B from 11.5-11.6 min; and 2% B held from 11.6-15 min for a final re-equilibration. The mass spectrometry was set to scan under the positive mode with the sheath gas, auxiliary gas, and sweep gas set to flow rates of 50, 13, and 3 psi, respectively. With the spray voltage set to 3.5 kV, the capillary and auxiliary gas heating temperature were respectively controlled to 263°C and 425°C to fully scan across *m/z* 70 to 1,000. The resolution was set to 70,000 FWHM (*m/z* 200). The automatic gain control (AGC) and the maximal injection time (MIT) was set to 2×10^5 and 50 msec, respectively. Routine mass calibrations were conducted before and after the sample analysis. The samples were blocked-randomized for the injection order. Quality control samples for the serum analytes were prepared by pooling the aliquots of each sample. Method blank samples were prepared for the serum samples by surrogating the biospecimen with water and following the same experimental procedures. If MS/MS spectrums were to be collected, the parallel reaction monitoring (PRM) mode was used, with the isolation width, AGC, and MIT set to 1.2, 3×10^5 and 100 msec, respectively, at the resolution of 17,500 FWHM (*m/z* 200). The quality and performance of the LC-MS analysis was assessed and ensured by monitoring the distribution of metabolite signal among samples (**Figure S3**) fluctuation of retention time and signal area of the spiked internal standards (**Figure S4**).

Data processing for LC-MS analysis. Data obtained from the LC-MS analyses were converted and centroided to mzXML files with ProteoWizard ver 3. The data processing of the fecal and serum metabolomic profiles of the samples were respectively composed of two stages: feature identification and compound characterization. For feature annotation, XCMS (ver 3.16.1) was used in coordination of the IPO package (ver 1.20.0) to optimize parameters, which the methods were referenced in past studies [8-12]. The data from the quality control samples were used to optimize the parameters in the algorithms of XCMS, including feature detection

(*centWave)*, retention time adjustment (*obiwarp*), and correspondence (*density*), using the IPO package. Then, all LC-MS data were processed with XCMS, optimized in parameters, to obtain a list of molecular features with distinct *m/z*-RT signatures, and only features detected in 50% of the vehicle control, low-dose, or high-dose group were kept for subsequent analysis. Missing values in the measurement matrix were imputated with the random forest algorithm by the missForest package (ver.1.4) as suggested in previous comparison studies [13-15]. The features were further analyzed for the pattern and coherence of isotopes and adducts, and matched with our in-house LC-MS library by Compound Discoverer ver 3.3 (Thermo Fisher Scientific). Our in-house library was built by testing authenticated standards of 734 common metabolites, and the criteria for a reliable match was a difference lower than 30 seconds and 5 ppm in retention time and accurate mass, respectively. Feature-wise student t test and F test after log-transforming the data were applied for the differential analysis between $Fxr^{-/-}$ and wild-type (WT) mice, and the p-values were adjusted by the Benjamin-Hochberg method (q-values). The (strict) criteria to determine significant difference between the $Fxr^{-/-}$ and wild-type (WT) mice in serum metabolites were 1.5-fold-change in either directions and $q < 0.05$.

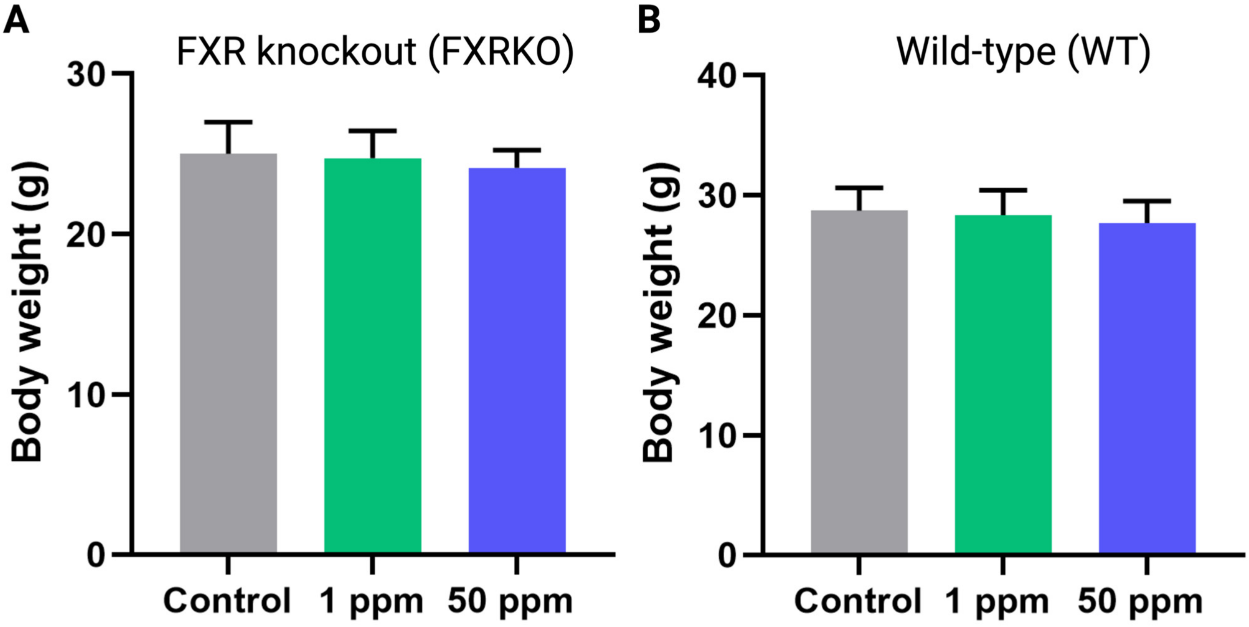

Figure S1. FXR knockout (FXRKO) mice developed lower body weight in general compared to wild-type (WT) mice. (A) Body weight for FXRKO mice. (B) Body weight for WT mice.

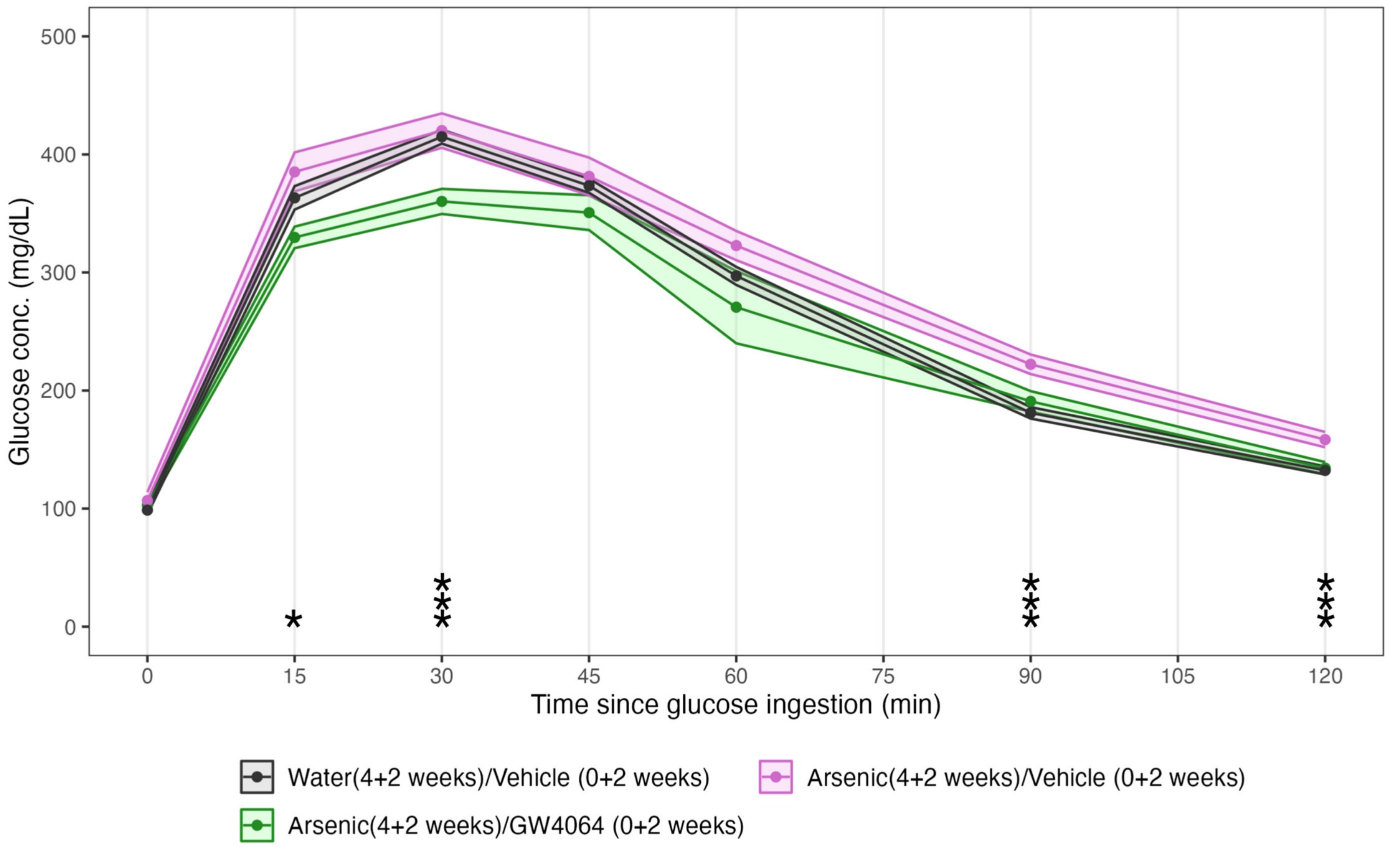

Figure S2. Blood glucose level throughout glucose tolerance test for the second stage of animal experiments. (A) Blood glucose dynamics of the mice in Groups A (Clean water, no GW4064 supplement, grey), B (Water with arsenic, no GW4064 supplement, orchid), and C (Water with arsenic, with GW4064 supplement, green). * $p < 0.05$, ** $p < 0.01$, *** $p < 0.001$ in the Student *t* test.

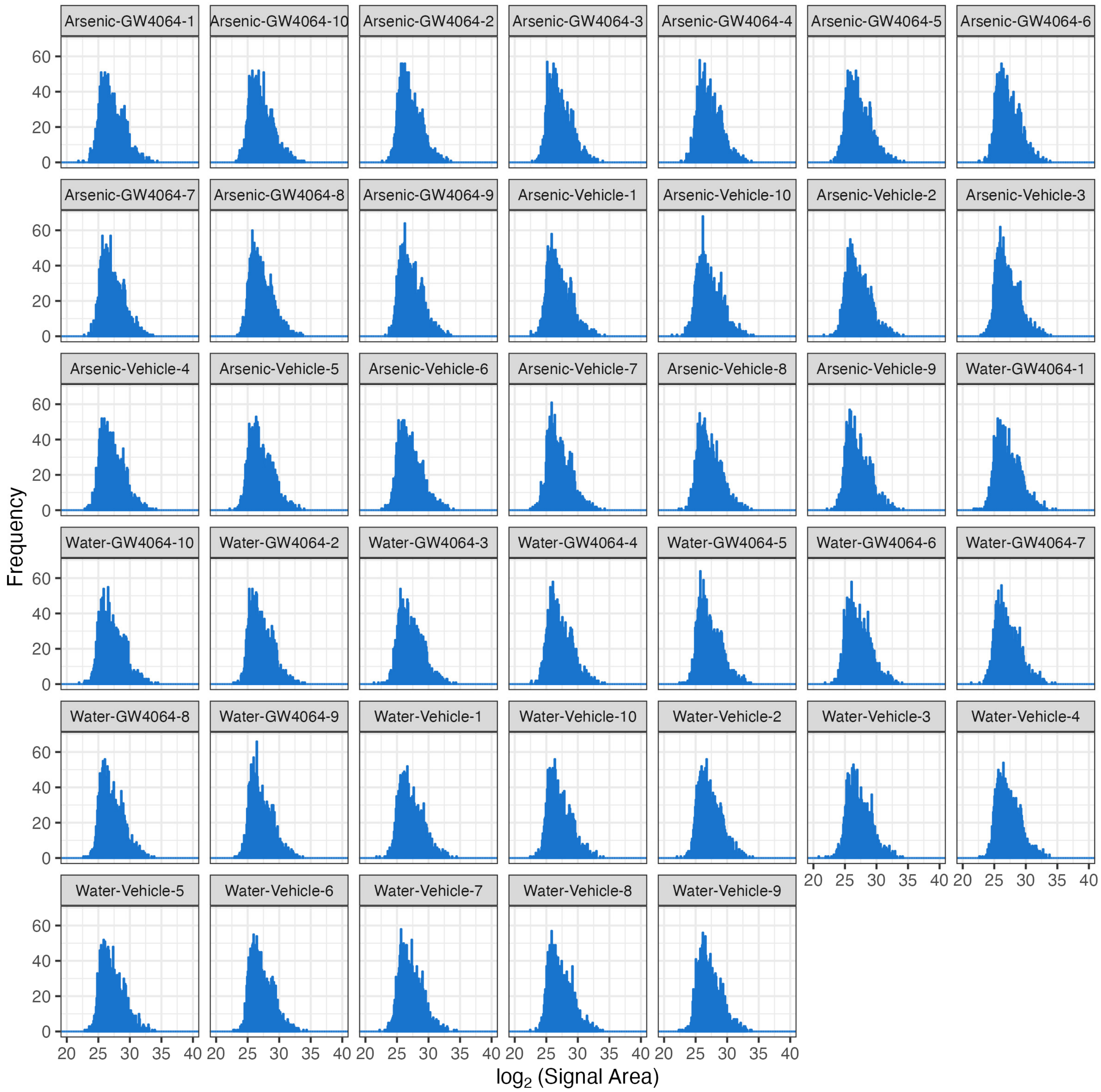

Figure S3. Signal distribution of the label-free quantitation of the hepatic proteome by nanoLC-nanoESI-MS/MS for the four groups of mice in the second stage of animal experiments.

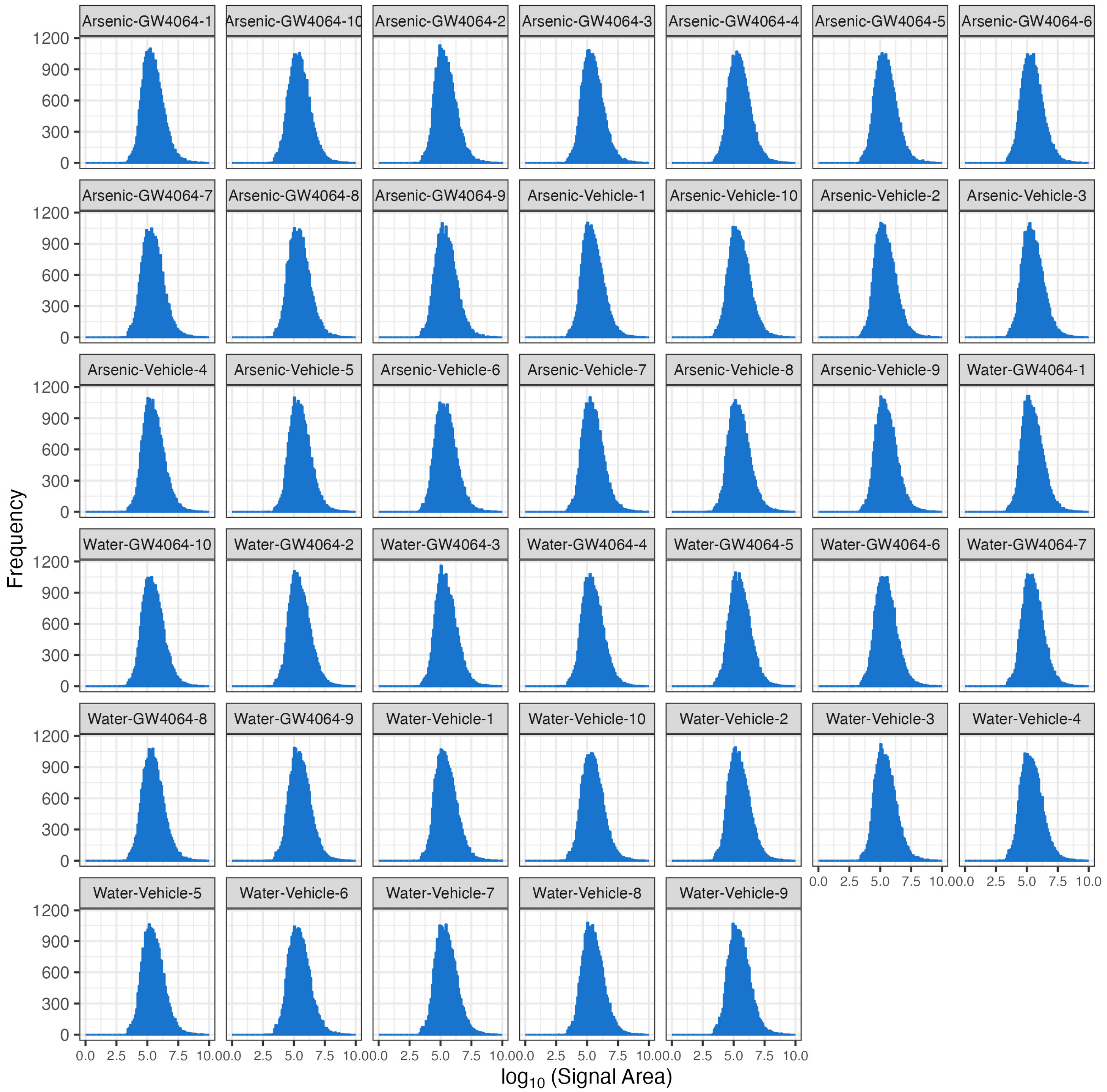

Figure S4. Signal distribution of the nontargeted serum metabolomic by LC-MS for the four groups of mice in the second stage of animal experiments.

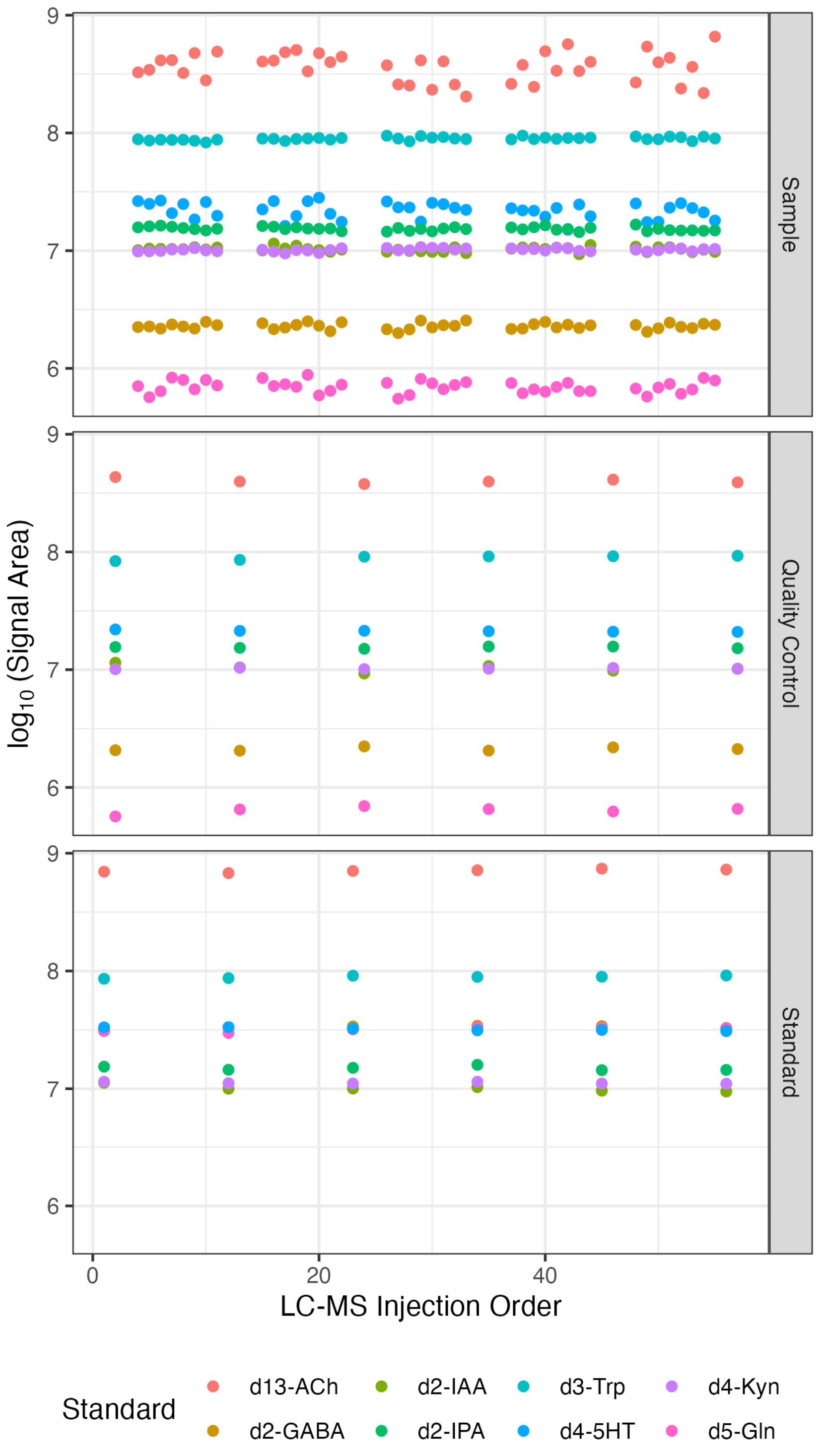

Figure S5. Signal areas of the stable isotope-labeled standards spiked in each sample. Most of spiked standards showed high consistency in signal areas. The signal areas of [d_5]-glutamine and [d_{13}]-acetylcholine had higher variation due to its very high hydrophilicity (~ 0.6 and 0.7 minutes in retention time, respectively). Ach: acetylcholine, IAA: indole-3-acetate, Trp: tryptophan, Kyn: kynurenine, GABA: γ-aminobutyric acid, IPA: indole-3-propionate, 5HT: serotonin, Gln: glutamine.

References

1. Zhang, L.; Liu, C.-W.; Zhang, Q. Online 2D-LC-MS/MS Platform for Analysis of Glycated Proteome. *Anal. Chem.* **2018**, *90*, 1081–1086. https://doi.org/10.1021/acs.analchem.7b03342.
2. Liu, C.-W.; Zhang, Q. Isobaric Labeling-Based LC-MS/MS Strategy for Comprehensive Profiling of Human Pancreatic Tissue Proteome. *Methods Mol. Biol.* **2018**, *1788*, 215–224, doi:10.1007/7651_2017_77.
3. Tyanova, S.; Temu, T.; Cox, J. The MaxQuant computational platform for mass spectrometry-based shotgun proteomics. *Nat. Protoc.* **2016**, *11*, 2301–2319. https://doi.org/10.1038/nprot.2016.136.
4. Tyanova, S.; Temu, T.; Sinitcyn, P.; Carlson, A.; Hein, M.Y.; Geiger, T.; Mann, M.; Cox, J. The Perseus computational platform for comprehensive analysis of (prote)omics data. *Nat. Methods* **2016**, *13*, 731–740. https://doi.org/10.1038/nmeth.3901.
5. Lai, Y.; Liu, C.-W.; Yang, Y.; Hsiao, Y.-C.; Ru, H.; Lu, K. High-coverage metabolomics uncovers microbiota-driven biochemical landscape of interorgan transport and gut-brain communication in mice. *Nat. Commun.* **2021**, *12*, 6000. https://doi.org/10.1038/s41467-021-26209-8.
6. Guo, H.; Chou, W.-C.; Lai, Y.; Liang, K.; Tam, J.W.; Brickey, W.J.; Chen, L.; Montgomery, N.D.; Li, X.; Bohannon, L.M.; et al. Multi-omics analyses of radiation survivors identify radioprotective microbes and metabolites. *Science* **2020**, *370*, eaay9097 https://doi.org/10.1126/science.aay9097.
7. Hsiao, Y.-C.; Liu, C.-W.; Robinette, C.; Knight, N.; Lu, K.; Rebuli, M.E. Development of LC-HRMS untargeted analysis methods for nasal epithelial lining fluid exposomics. *J. Expo. Sci. Environ. Epidemiol.* **2022**, *32*, 847–854. https://doi.org/10.1038/s41370-022-00448-3.
8. Cheema, A.K.; Li, Y.; Girgis, M.; Jayatilake, M.; Fatanmi, O.O.; Wise, S.Y.; Seed, T.M.; Singh, V.K. Alterations in Tissue Metabolite Profiles with Amifostine-Prophylaxed Mice Exposed to Gamma Radiation. *Metabolites* **2020**, *10*, 211. https://doi.org/10.3390/metabo10050211.
9. Fernández-Ochoa, Á.; Quirantes-Piné, R.; Borrás-Linares, I.; Cádiz-Gurrea, M.d.l.L.; PRECISESADS Clinical Consortium; Riquelme, M.E.A.; Brunius, C.; Segura-Carretero, A. A Case Report of Switching from Specific Vendor-Based to R-Based Pipelines for Untargeted LC-MS Metabolomics. *Metabolites* **2020**, *10*, 28. https://doi.org/10.3390/metabo10010028.
10. Libiseller, G.; Dvorzak, M.; Kleb, U.; Gander, E.; Eisenberg, T.; Madeo, F.; Neumann, S.; Trausinger, G.; Sinner, F.; Pieber, T.; et al. IPO: A tool for automated optimization of XCMS parameters. *BMC Bioinform.* **2015**, *16*, 118. https://doi.org/10.1186/s12859-015-0562-8.
11. Tautenhahn, R.; Böttcher, C.; Neumann, S. Highly sensitive feature detection for high resolution LC/MS. *BMC Bioinform.* **2008**, *9*, 504–504. https://doi.org/10.1186/1471-2105-9-504.
12. Smith, C.A.; Want, E.J.; O'Maille, G.; Abagyan, R.; Siuzdak, G. XCMS: Processing Mass Spectrometry Data for Metabolite Profiling Using Nonlinear Peak Alignment, Matching, and Identification. *Anal. Chem.* **2006**, *78*, 779–787. https://doi.org/10.1021/ac051437y.
13. Kokla, M.; Virtanen, J.; Kolehmainen, M.; Paananen, J.; Hanhineva, K. Random forest-based imputation outperforms other methods for imputing LC-MS metabolomics data: A comparative study. *BMC Bioinform.* **2019**, *20*, 492. https://doi.org/10.1186/s12859-019-3110-0.
14. Stekhoven, D.J.; Bühlmann, P. MissForest—Non-parametric missing value imputation for mixed-type data. *Bioinformatics* **2011**, *28*, 112–118. https://doi.org/10.1093/bioinformatics/btr597.
15. Wei, R.; Wang, J.; Su, M.; Jia, E.; Chen, S.; Chen, T.; Ni, Y. Missing Value Imputation Approach for Mass Spectrometry-based Metabolomics Data. *Sci. Rep.* **2018**, *8*, 663. https://doi.org/10.1038/s41598-017-19120-0.
16. Vinaixa, M.; Samino, S.; Saez, I.; Duran, J.; Guinovart, J.J.; Yanes, O. A Guideline to Univariate Statistical Analysis for LC/MS-Based Untargeted Metabolomics-Derived Data. *Metabolites* **2012**, *2*, 775–795. https://doi.org/10.3390/metabo2040775.
17. Chen, Y.; Li, E.-M.; Xu, L.-Y. Guide to Metabolomics Analysis: A Bioinformatics Workflow. *Metabolites* **2022**, *12*, 357. https://doi.org/10.3390/metabo12040357.
18. Brunnsåker, D.; Reder, G.K.; Soni, N.K.; Savolainen, O.I.; Gower, A.H.; Tiukova, I.A.; King, R.D. High-throughput metabolomics for the design and validation of a diauxic shift model. *Npj Syst. Biol. Appl.* **2023**, *9*, 11. https://doi.org/10.1038/s41540-023-00274-9.
